# The Medical Schools

**Published:** 1905-09-02

**Authors:** 


### The Medical Schools.

The choice of a medical school is a matter that should receive grave attention from the parents, guardians, or advisers of intending medical students. Medical schools vary considerably in their tone, in their traditions, in the quality of instruction provided, and in many other ways, and the ultimate success in life of an intending medical practitioner may hang to a large extent on the choice of his school.

While London will probably always occupy the leading place in the United Kingdom as a centre of medical education, it does not follow that it is invariably the best place for a prospective medical student. Several of the large provincial towns possess flourishing medical schools and can claim as their children some of the leading men in the profession, and, indeed, they possess many advantages which are not found in the metropolis. The size of a hospital is not of great importance for teaching purposes; of more consequence is the size of the medical school. Other things being equal, a large school is usually better than a small one, because it is better able to supply well equipped laboratories and first-rate teachers.

It is impossible to generalise much further than this on the question of the choice of a school. Individual requirements are probably the surest guides.

Under the head "composition fee" we have mentioned the sum required by each hospital and medical school for all the classes, lectures, and hospital attendance which are necessary for the ordinary diplomas, but at each of the hospitals special arrangements can be made for separate courses.

All that is here attempted is to give a more or less general impression of the various medical schools; for further information and particulars application should be made to the individual institutions by letter addressed to "The Dean of the Medical School."

### METROPOLITAN MEDICAL SCHOOLS.

#### St. Bartholomew's Hospital Medical School.

St. Bartholomew's is the oldest and second largest hospital in London, and is, perhaps, the leading medical school. The site has been recently enlarged by the purchase of 1½ acre of adjoining ground, and the first block of new buildings is in course of erection at a cost of £120,000. It will contain new casualty and out-patient departments, eight special departments, new quarters for the resident staff, a dining-hall and common room for students, etc. Two additional operation theatres have been built, making seven in all. The erection of this block will not interfere with the work either of the hospital or the school.

The hospital contains 670 beds, and there is therefore an abundance of clinical material. Several entrance scholarships are awarded annually, and the total value of scholarships and prizes offered every year amounts to nearly £900. There is a college for resident students within the precincts of the hospital, for admission to which a recommendation from one of the medical officers of the hospital is required. St. Bartholomew's is a constituent school of the University of London, and the preliminary subjects —chemistry, physics, and biology—are taught, as well as those for the intermediate and final examinations. Special classes are held for students taking the degrees of the older Universities or special diplomas in surgery and public health.

There is an entrance fee of 30 guineas and an annual fee of 30 guineas. The entrance fee and the five annual fees for the entire curriculum can be commuted by a single payment of 165 guineas University students who have completed their anatomical and physiological studies pay an entrance fee of 20 guineas only and two annual fees. On qualification at the end of the time covered by the annual fees a student receives a perpetual ticket, but should he fail to qualify in these periods there is a fee of 10 guineas for each further six months' instruction.

### Charing Cross Hospital Medical School.

Situated in the very centre of London, this hospital has perhaps an advantage over all other similar institutions in the metropolis as regards accessibility.

Extensive additions have recently been made, including new surgical wards, out-patient department, operating theatres, and isolation wards.

The medical school occupies a separate building in Chandos Street, immediately opposite to the hospital.

Several entrance scholarships are offered annually for competition. Missionary studentships are also obtainable by clergymen through the Society for the Promotion of Christian Knowledge.

The composition fee—including subscription to the students' club—is £115.

### St. George's Hospital Medical School.

The hospital contains 350 beds, of which 205 are devoted to surgical and 146 to medical cases.

There are several entrance and other scholarships.

The entire laboratories and teaching at the hospital and school are now devoted to the essentially medical subjects. Unequalled facilities are therefore available for clinical instruction and research.

There are numerous appointments open to the senior students, and amongst others may be specially mentioned the following paid appointments, which are open to students after they have held house office :—

| | | | |
|---|---|---|---|
| Two Medical Registrarships ... | salary | £200 | per annum |
| Surgical Registrarship ... ... | ,, | 200 | ,, |
| Curatorship of the Museum ... | ,, | 200 | ,, |
| Assistant Curatorship ... ... | ,, | 100 | ,, |
| Resident Obstetric Assistantship ... | ,, | 50 | ,, |
| Two Demonstratorships of Anatomy | ,, | 50 | ,, |
| Senior Anæsthetists... ... ... | ,, | 50 | ,, |
| Two Junior Anæsthetists ... ... | ,, | 30 | ,, |

### Guy's Hospital Medical School.

Guy's Hospital contains 602 beds, and a noteworthy feature is the reservation of certain wards, containing 40 beds in all, for cases of special clinical interest.

There is a college which accommodates about 60 students, who are under the supervision of a resident warden. The rooms are furnished, and the cost of residence is moderate.

By paying an annual subscription of two guineas any student of the hospital obtains the privileges of membership of all the clubs, including the students' club.

Situated on the south side of the river near London Bridge, the hospital ministers to a very large population; there is, therefore, plenty of work to be done, and the house appointments are especially valuable.

The composition fees are 16 guineas for the first twelve months or less period, and subsequently 30 guineas a year till the student is qualified.

### King's College Hospital.

King's College Hospital, which contains 220 beds, is situated in Portugal Street, Lincoln's Inn, and is about five minutes' walk from King's College.

The new hospital, which is now being built in the neighbourhood of Denmark Hill, will be on a much larger scale than the present institution, and will possess every modern requirement for both patients and students. The teaching of the preliminary subjects, including chemistry, physics, biology, anatomy, and physiology, will continue to be given at King's College in the Strand, and students entering now may by the time they are sufficiently advanced to study in the wards be able to do so the new hospital.

Several entrance scholarships are offered for competition every year, including two of the value of £25 per annum, each tenable for four years, and one of £100. There are several others of smaller value. Medical missionary studentships are also obtainable through the Society for Promoting Christian Knowledge.

The composition fee is—for University course, 140 guineas; for conjoint course, 135 guineas. Full particulars and prospectuses can be obtained by applying to Professor Halliburton, Dean of the Medical Division of the Faculty of Science, or Dr. Whitfield, Dean of the Hospital.

### The London Hospital Medical School.

The London Hospital is the largest institution of its kind in the metropolis, and being situated in the East End, it ministers to a very large and poor district.

Several entrance scholarships are offered annually, including the Price Scholarship in science, £120; the Epsom Scholarship, £126; and entrance scholarships in science, anatomy and physiology, and arts

There is a Clubs' Union, embracing the athletic and all the other clubs, to which all students are expected by the College Board to belong. The recreation ground is at Highams Park, and special arrangements have been made with the Great Eastern Railway for tickets at reduced rates for members of the union.

The composition fee is 120 guineas if paid in one sum, or 130 guineas if paid in three annual instalments. A reduction of 15 guineas is allowed in the case of sons of medical men.

Full information may be obtained from the Warden of the Medical School.

### St. Mary's Hospital Medical School.

St. Mary's Hospital at present contains 281 beds, which number will be raised to 341 on the opening of the Clarence Wing, which will shortly be ready for occupation. This extension will also provide new operating theatres, a medical clinical theatre, an obstetric department for cases of difficult labour, and a complete electrical and *x*-ray department.

Six entrance scholarships in natural science are offered annually, and include one of £145 open to any student who has not completed a Winter Session of medical study at a medical school, two of 60 guineas each open to students of any British University, and one of £145 open to students from Epsom College, being the sons of medical men, who have not completed a Winter Session of study at a medical school. This scholarship is not awarded [illegible] examination.

The composition fee is £140 if paid in one sum on entering the Medical School, and £145 if paid in four instalments. On payment of the full composition fee, and provided that he obtains a registrable medical qualification within seven years from the date of registration, the student becomes a perpetual pupil, and is entitled to unlimited attendance on the practice of the hospital. The composition fee for students who have passed their examination in anatomy and physiology is 60 guineas if paid at entrance, 65 guineas if paid in two annual instalments.

### The Middlesex Hospital Medical School.

The hospital, which is in Mortimer Street, W., contains 340 beds, including special wards for children and the diseases of women. There is a cancer wing which has 40 beds, and special research laboratories, and the hospital therefore offers unequalled opportunities for the study of this disease, both clinically and pathologically.

A complete electrical and *x*-ray department has recently been provided in a temporary building which has been erected in the courtyard of the hospital.

The hospital has the advantage of possessing a residential college which faces on the hospital garden. It is capable of accommodating about 30 students.

There is an amalgamated students' club, which all general and dental students are required to join on their entrance to the medical school.

Several entrance and other scholarships and prizes are offered annually.

The composition fee, if paid in one sum on entrance, is 135 guineas. It may be paid, if preferred, in three instalments, viz.: On entrance, 60 guineas; at commencement of second year, 50 guineas; at commencement of third year, 35 guineas.

### St. Thomas's Hospital Medical School.

St. Thomas's Hospital contains 602 beds, of which about 540 are in constant use.

There are numerous scholarships and prizes in connection with the school.

The various students' clubs were amalgamated in 1888. The annual subscription for membership of the amalgamated clubs is £2 10s. for the first three years and 2 guineas for the years following. After five consecutive subscriptions the student becomes a life-member, provided he has obtained a registrable qualification.

The composition fee covering the work of the first year is 20 guineas. Students who have passed the preliminary scientific or a corresponding examination pay an entrance fee of 20 guineas and an annual fee of 30 guineas, which is due in advance on the first day of the term in which the student enters, and on the corresponding day of each successive year until a registrable qualification has been obtained. A student who qualifies within six months of the date on which his last annual composition fee became due is allowed a rebate of 15 guineas.

The entrance fee to students who have passed the intermediate M.B.Lond. or a corresponding examination is 10 guineas. The annual composition fee until qualification is 30 guineas.

### University College, London (Faculty of Medicine), and University College Hospital.

University College, like King's College, is completely equipped for providing educational facilities in a wide range of subjects, of which medicine is one. The medical school is very flourishing, and takes a high place among its fellow-institutions in London.

Scholarships and exhibitions of the value of £800 are offered for competition every year.

Composition Fees:—For the courses required by the University of London, 165 guineas, payable as follows: For the preliminary scientific course, 25 guineas; for the intermediate, 60 guineas, or in two instalments of 37 and 25 guineas; and for the final M.B. course, 80 guineas, or in two instalments of 50 and 32 guineas.

For the medical education required by the Examining Board in England and the Society of Apothecaries:—For the course required for the first examination, 30 guineas, entitling to one attendance. For the course required for the second examination, 50 guineas (if paid in one sum), 51 guineas (if paid in two instalments), as follows: First year, 31 guineas; second year, 20 guineas (this fee entitles to attendance during three years). For the course required for the third examination, 80 guineas (if paid in one sum), 82 guineas (if paid in two instalments), as follows: First year, 50 guineas; second year, 32 guineas.

### Westminster Hospital Medical School.

Westminster Hospital was the first institution of its kind in London which was founded by the voluntary contributions of the public.

There is accommodation for upwards of 200 in-patients.

There is a Students' Club Union, the subscription for membership of which is included in the general fees.

The composition fee is 110 guineas for the course of the Conjoint Board, and 133 guineas for that of the University of London.

### London (Royal Free Hospital) School of Medicine for Women.

The Royal Free Hospital is in Gray's Inn Road, and the London School of Medicine for Women is in Hunter Street, Brunswick Square, W.C.

There are 170 beds in the hospital, all of which are available for clinical teaching.

There are numerous scholarships and prizes offered annually in connection with the school, among which may be mentioned the School Scholarship of £30 and the St. Dunstan's Medical Exhibition of £60 a year for three years, extendible to five years.

The composition fee for the entire course after the Preliminary Scientific Examination for the M.B. of the London University and the Royal University of Ireland is £135, and that for the Preliminary Scientific Course is 25 guineas.

The composition fee for the course for the M.B. of the University of Durham, for the diploma of the Conjoint Colleges of Scotland, or the Society of Apothecaries, including Elementary Science, is £140.

---

### PROVINCIAL MEDICAL SCHOOLS.

The provincial medical schools at which a complete medical education can be obtained are those of Birmingham, Bristol, Cambridge, Newcastle (University of Durham), Leeds, Liverpool, Manchester, and Sheffield. Certificates of attendance at any of these schools are accepted by the Royal Colleges, and also by the University of London.

### The University of Birmingham (Faculty of Medicine).

Candidates are allowed to enter for the First Professional M.B., Ch.B. Examination (Chemistry and Physics) before commencing residence in the University, provided that they have already passed the Matriculation Examination or some preliminary examination accepted by the University in lieu of its matriculation, such candidates to be eligible for the final examination for M.B., Ch.B., after four years' residence in the University and by producing evidence that they have been registered medical students by the General Medical Council for a period of five years.

In order to obtain the degrees of M.B. and Ch.B. five examinations have to be passed. The first is in chemistry and physics; the second in anatomy, comparative anatomy, and physiology; the third in pathology, bacteriology, and materia medica and pharmacy; the fourth in forensic medicine, toxicology, and public health; and the fifth, or final, in medicine, surgery, midwifery, gynæcology, therapeutics, mental diseases, and ophthalmology. The University also grants a diploma (L.D.S.) and degrees in dental surgery and a diploma and a degree of B.Sc. in public health.

The General Hospital and the Queen's Hospital, containing between them upwards of 400 beds, are amalgamated for the purpose of clinical instruction, which is carried on under the direction of the Birmingham Clinical Board.

The composition fee for the whole course of lectures and instruction at the University is £85, besides which there is a composition fee for attendance on the medical and surgical practice and the clinical lectures at both hospitals of £42, making a total of £127.

### University College (Faculty of Medicine), Bristol.

Students of the college are admitted to the clinical practice of the Bristol Royal Infirmary and the Bristol General Hospital conjointly. The infirmary and the hospital comprise between them a total of 470 beds, and both are provided with extensive out-patient departments and special departments. The practice of the Bristol Royal Hospital for Sick Children and Women, which contains 104 beds, and that of the Bristol Eye Hospital, with 40 beds, are also available.

The total number of beds open for clinical instruction is therefore 614.

The composition fee for the lectures is 70 guineas, and that for hospital practice is 35 guineas.

Special fees are charged for clerkships and dresserships, and the total fees payable, including these, amount to 132 guineas.

### University of Cambridge.

In order to obtain the degree of Bachelor of Medicine a student must become a matriculated student of the University, reside in the University the required portion of each of nine terms, pass or obtain exemption from the previous examination, pursue medical study for five years, and pass three examinations.

Instruction in clinical medicine, surgery, and midwifery is given at Addenbrooke's Hospital, so that it is possible to obtain a complete medical education at Cambridge; but the usual practice is for men, after spending three or four years at the University, and passing their examinations in anatomy and physiology, and in pharmacology and general pathology, to finish their clinical work in London or one of the large provincial towns.

### University College, Cardiff.

Since it was founded, in 1883, University College, Cardiff, has prepared students for the Preliminary Scientific examination of the University of London, and for the corresponding examinations of other licensing bodies. In 1893, Chairs of Anatomy and Physiology and a Lectureship in Materia Medica and Pharmacy were established, making it possible to spend three out of the five years of the medical curriculum at this school.

Arrangements have been made with the managing committee of the Cardiff Infirmary to give students of the college the privilege of attending this hospital, which contains 180 beds.

The composition fee for the three years' course of study for the Preliminary Scientific and Intermediate Examination for the London M.B. is £57 10s.

### University of Durham (College of Medicine, Newcastle-upon-Tyne).

For students who intend to graduate at the Durham University it is necessary that one of the five years of professional education should be spent in attendance at the College of Medicine, Newcastle-upon-Tyne. During the year so spent the student must attend the medical and surgical practice and clinical lectures at the Infirmary, which contains 280 beds. No residence at Durham is necessary. The remaining four years of the curriculum may be spent at Newcastle-upon-Tyne or at one or more of the recognised medical schools.

A new wing is in course of erection at the College of Medicine which will accommodate the departments of physiology and bacteriology and contains a students' gymnasium and a set of students' union rooms and will be completed by the summer of 1906.

The New Royal Victoria Infirmary, containing 450 beds, is approaching completion, and will be opened in the course of next summer. In the new infirmary adequate accommodation will be provided for the study of the various special subjects in addition to the ordinary clinical work.

The composition fee for the complete course of lectures is 72 guineas, and for the medical and surgical practice of the hospital 25 guineas.

### The University of Leeds (School of Medicine).

Clinical instruction is given in the general infirmary, which contains 480 beds, and is amply provided with special departments.

Students of the University also have access for instruction to the Leeds Public Dispensary, the City Fever Hospitals, the Hospital for Women and Children, and the West Riding Asylum at Wakefield.

The School of Medicine, which is particularly well equipped in every way, is separated from the infirmary by the width of a street only, an arrangement which facilitates attendance at both institutions.

The composition fee for university students who have already taken the course of instruction for the first M.B. examination, and for other students who have taken out the required instruction in chemistry, chemical physics, and elementary biology, is 64 guineas, and that for the hospital practice is 40 guineas.

### The University of Liverpool Faculty of Medicine.

Students are prepared for the degrees of the University of Liverpool, or may study for the degrees and qualifications of various other licensing bodies.

The laboratories are modern and very well equipped, and the facilities for instruction are most complete.

The clinical work may be done at the Royal Infirmary, which contains 300 beds, or at the United Hospitals' Clinical School, consisting of the David Lewis Northern Hospital, where there are 200 beds, the Royal Southern Hospital containing 200 beds, or the Stanley Hospital with 104 beds.

The lying-in, eye and ear, children's, and other special hospitals are also open to students, and courses of instruction in tropical diseases can be obtained at the Liverpool School of Tropical Medicine. Fellowships, scholarships, and prizes of over £800 are awarded annually.

### The Victoria University of Manchester (Faculty of Medicine).

The medical department of the University is particularly well provided with facilities for teaching the preliminary subjects—namely, anatomy, physiology, pathology, and materia medica.

Clinical instruction is provided at the Manchester Royal Infirmary, which contains 290 beds, and also has large out-patient and casualty departments. Associated with the Infirmary is a Convalescent Hospital at Cheadle, containing 136 beds. Women students are admitted to the practice of the Infirmary on the same terms as men.

Three halls are licensed by the college for the residence of students — namely, Dalton Hall, Victoria Park, and Hulme Hall, Plymouth Grove, for men, and Ashburne House, Victoria Park, for women.

The composition fee for the full course of medical study required for the ordinary qualifications is £80, in addition to which the fee for hospital practice is £42.

Further particulars may be obtained from the Dean of the Medical Faculty.

### Oxford University.

One of the chief events in the recent history of Oxford University has been the revival of the Medical School. Admirable arrangements now exist for instruction in the preliminary medical sciences; while a certain proportion of the clinical work can be done, if desired, in the wards of the Radcliffe Infirmary, previous to taking out the full course in the metropolis. Clinical lectures are given in medicine and surgery, and demonstrations in pathology, surgical anatomy, and medical and surgical diagnosis are given also. In order to obtain the Oxford medical degree it is necessary to be a graduate in Arts of the University.

### University of Sheffield (Faculty of Medicine).

The University is within easy distance of the Royal Infirmary, the Royal Hospital, the Jessop Hospital for Women, and the City Fever Hospital. It possesses numerous well-equipped laboratories and other necessaries for study and research. There is a large field for clinical work in the above-named hospitals.

The composition fee is £90, which admits to classes and courses of instruction required for the M.B., Ch.B. degrees of the University. This fee does not cover medical and surgical hospital practice and clinical work. An additional fee of £45 will entitle a student to a perpetual ticket for hospital practice, excepting that of the Jessop Hospital, the Fever Hospital, and the South Yorkshire Asylum, the fees for which must be paid separately.

---

### SCOTTISH MEDICAL SCHOOLS.

### University of Edinburgh.

To obtain the Edinburgh medical degree it is necessary to pass the Preliminary Examination or its recognised equivalent, and to pass four professional examinations. The first is in botany, zoology, physics, and chemistry; the second in anatomy, physiology, materia medica, and therapeutics; the third in pathology; and the final in surgery, clinical surgery, medicine, clinical medicine, midwifery, forensic medicine, and public health.

The degree of M.B. is not conferred separately from the Ch.B. In order to obtain the degree it is necessary to spend at least two of the five years of medical study in the University of Edinburgh.

Clinical instruction is given in the Royal Infirmary, which contains nearly 900 beds; Royal Hospital for Sick Children, with 120 beds; Maternity Hospital; City Fever Hospital; and the Royal Morningside Asylum. The Faculty of Medicine has recently resolved upon the institution of a diploma in Tropical Medicine and Hygiene, and has prepared a scheme of education and examination for this diploma.

### School of Medicine of the Royal Colleges, Edinburgh.

This School of Medicine is composed of lecturers who lecture in several buildings near the Royal Infirmary. They are recognised or licensed by the Royal Colleges of Surgeons and Physicians, and also recognised by the University.

The teaching is similar to that of the Scottish Universities, and the students receive similar certificates at the close of each session. The lectures qualify for the University of Edinburgh and other Universities; the Royal Colleges of Physicians and Surgeons of Edinburgh, London and Dublin; the Faculty of Physicians and Surgeons of Glasgow, and the other Medical Boards. The whole Education required for graduation at the University of London may be taken in this school.

The facilities for clinical instruction are similar to those provided for the University.

### Medical College for Women, Edinburgh.

Precisely the same facilities for medical study are given as to male students in the School of Medicine, Edinburgh. Regulations, fees, and arrangements are the same. The lecturers are recognised by the University of Edinburgh as giving courses admitting to the degrees of M.B., Ch.B. Clinical instruction is given in the Royal Infirmary and other hospitals mentioned above.

The benefit of the Carnegie Trust is open to students of the college.

### University of Glasgow.

The whole course of study for the M.B., Ch.B., can be passed in the Medical School of the University.

The regulations are similar to those given above for Edinburgh. Clinical instruction is given at the Western Infirmary, which contains 420 beds, and also at the Lunatic Asylum, the Eye Infirmary, the Maternity Hospital, the City Fever Hospitals, and various other hospitals.

The cost of the course, including matriculation, fees for classes, for hospital attendance, and for professional examinations, amounts to about £150.

### Queen Margaret College, Glasgow (The Women's Department of the University).

This is an integral part of the University of Glasgow. The courses, regulations, and fees are the same as for men. The instruction is given by University Professors and Lecturers appointed by the University Court. The college provides a separate building, with class-rooms, laboratories, library, and other teaching appliances. The women have all the rights and privileges of University students, and do their clinical work in the Royal Infirmary, where special wards are reserved for their use, and in other hospitals.

### St. Mungo's College, Glasgow.

The college buildings, which are provided with class-room and laboratory accommodation for three or four hundred students, are situated within the grounds of the Royal Infirmary, the wards of which are available for clinical instruction, as also are those of the Western Infirmary. In connection with the former there are pathological and bacteriological laboratories, and a large pathological museum. All the classes in this college qualify for the diplomas of the English, Scottish, and Irish Conjoint Boards, and under certain conditions for the various universities.

### Anderson's College Medical School, Glasgow.

The college, at which a full medical curriculum can be obtained, is situated in Dumbarton Road, close to the Western Infirmary and within four minutes' walk of the University.

Degrees and diplomas: Certificates of attendance on the lectures are accepted by the Universities of London and Durham, by the Royal University of Ireland, and by all the Royal Colleges and licensing boards in the United Kingdom.

The Carnegie Trust extends its benefactions to students of Anderson's College. Particulars may be obtained from W. S. McCormick, Esq., LL.D., The Carnegie Trust Offices, Edinburgh.

### University of Aberdeen.

The regulations are practically the same as those given for Edinburgh. Clinical instruction is obtained in the Royal Infirmary, which contains 250 beds, in the Sick Children's Hospital, and several other institutions, including the Royal Lunatic Asylum and the City Fever Hospital.

### University of St. Andrews (University College, Dundee, and United College, St. Andrews).

In order to graduate, at least two of the five years of medical study must be spent either at the United College, St. Andrews, or at University College, Dundee. The remaining three years may be passed at other medical schools, but the whole course may be completed, if preferred, at University College, Dundee.

New medical buildings have been erected and equipped at University College, Dundee, at a cost for building and fittings of over £20,000, to give full space and equipment for the systematic and practical teaching chiefly of Physiology, Anatomy, Pathology, and Pharmacy and Therapeutics. Accomodation is also provided for the teaching of Surgery, Medicine, Forensic Medicine and Public Health, Ophthalmology, Mental Diseases, Midwifery, and Gynæcology.

---

### IRISH MEDICAL SCHOOLS.

### Royal College of Surgeons in Ireland (The Schools of Surgery).

These schools are attached by charter to the Royal College of Surgeons. They are carried on within the college buildings, and are specially subject to the supervision and control of the Council, who are empowered to appoint and remove the professors, and to regulate the methods of teaching pursued. The buildings have been reconstructed, the capacity of the dissecting-room nearly trebled, and special pathological, bacteriological, public health, chemical, and pharmaceutical laboratories fitted with the most approved appliances, in order that students may have the advantage of the most modern methods of instruction. There are special rooms set apart for lady students.

All the lectures and courses of practical instruction may be attended by medical students who are otherwise unconnected with the college.

A medical students' guide will be forwarded post free on application to the Registrar, Royal College of Surgeons, Dublin.

### The School of Physic in Ireland, Dublin.

This school is formed by an amalgamation of the medical school of Trinity College and of the Royal College of Physicians.

Clinical instruction is given by the staff of Sir Patrick Dun's Hospital, and courses of clinical study can be carried out at many of the other hospitals in Dublin.

### The Catholic University Medical School.

Complete courses of lectures, as required by all the examining bodies in the United Kingdom are provided.

For all further information about the school application should be made to the Registrar, Medical School, Cecilia Street, Dublin.

Queen's College, Belfast.

The courses of study meet the requirements of the Royal University of Ireland and other examining bodies.

Clinical instruction is given at the Royal Victoria Hospital and other hospitals.

Full information can be had on application to the Registrar, Queen's College, Belfast.

Queen's College, Cork.

Students can attend courses which meet the requirements of the Royal University of Ireland, the Conjoint Boards of London, Edinburgh, or Dublin, and other examining bodies.

Clinical instruction is given at the North and South Infirmaries, each of which contains 100 beds, and at other hospitals.

Further information can be had on application to the Registrar, Queen's College, Cork.

---

### MEDICAL SCHOOLS AND MEDICAL QUALIFICATIONS FOR WOMEN.

The following are the qualifying bodies which admit women as candidates for their degrees or diplomas:—all the Universities of England with the exception of the Universities of Oxford and Cambridge; the Universities of Scotland; the Royal Universities of Ireland; the Society of Apothecaries, London; and the Conjoint Board of the Royal Colleges of Physicians and Surgeons of Scotland and Ireland.

The regulations of the General Medical Council in regard to medical education and examinations apply to women exactly as they do to men.

With regard to place of study, women medical students can obtain a complete medical education by becoming students of the London (Royal Free Hospital) School of Medicine for Women, which is connected with the Royal Free Hospital; or Queen Margaret College, Glasgow (which is now an integral part of the University of Glasgow), both of which are open to women only; or they may study at the Medical College for Women, Edinburgh; the Schools of Surgery of the Royal College of Surgeons in Ireland, where special rooms are set apart for lady students; or at one of the Queen's Colleges at Belfast, Cork, and Galway. They can also enter at the Universities of Edinburgh, Aberdeen, and St. Andrews; at the Universities of Birmingham, Leeds, and Manchester, and at the University of Durham College of Medicine which is situated in Newcastle-upon-Tyne. The medical classes of the University of St. Andrews are open to women on an equal footing with men, and it may be arranged either to take the first two years at United College, St. Andrews, and the rest at Dundee, or the whole five years may be spent at University College, Dundee. The Dundee Royal Infirmary offers facilities for clinical instruction to women students.

For English women students who feel equal to the examinations of the London University, the London (Royal Free Hospital) School of Medicine for Women will be found thoroughly suitable, or they may spend a portion of their time at Newcastle after matriculating at the Durham University and then finish their course in London. The Secretary of the London (Royal Free Hospital) School of Medicine for Women, 8 Hunter Street, London, W.C., will give every information.

